# Survivorship care plans have a negative impact on long-term quality of life and anxiety through more threatening illness perceptions in gynecological cancer patients: the ROGY care trial

**DOI:** 10.1007/s11136-018-1825-4

**Published:** 2018-03-06

**Authors:** Belle H. de Rooij, Nicole P. M. Ezendam, Kim A. H. Nicolaije, Paul Lodder, M. Caroline Vos, Johanna M. A. Pijnenborg, Dorry Boll, Roy F. P. M. Kruitwagen, Lonneke V. van de Poll-Franse

**Affiliations:** 1 Department of Medical and Clinical Psychology, CoRPS - Center of Research on Psychology in Somatic diseases, Tilburg University, Warandelaan 2, PO Box 90153, 5000 LE Tilburg, The Netherlands; 2 The Netherlands Comprehensive Cancer Organisation, Utrecht, The Netherlands; 3 Department of Obstetrics and Gynecology, Gynecologic Cancer Center South, Elisabeth-TweeSteden Hospital, Tilburg and Waalwijk, The Netherlands; 4 Department of Obstetrics & Gynecology, Radboud University Medical Center, Nijmegen, The Netherlands; 5 Department of Gynecology, Catharina Hospital, Eindhoven, The Netherlands; 6 Department of Gynecology and GROW - School for Oncology and Developmental Biology, Maastricht University Medical Center, Maastricht, The Netherlands; 7 Division of Psychosocial Research and Epidemiology, The Netherlands Cancer Institute, Amsterdam, The Netherlands

**Keywords:** Survivorship care plan, Information provision, Gynecologic cancer, Illness perception, Quality of life, Anxiety

## Abstract

**Purpose:**

Prior results from the registration system oncological gynecology (ROGY) care trial showed that survivorship care plans (SCPs) increased threatening illness perceptions in gynecological cancer survivors, but it remained unclear whether this would result in poorer physical and psychosocial outcomes. The aim of the current study is to assess the direct and indirect effects of SCPs on health-related quality of life (HRQoL) and anxiety and depression, through illness perceptions.

**Methods:**

Twelve hospitals in the South of the Netherlands were randomized to providing ‘SCP care’ or ‘usual care.’ Newly diagnosed endometrial and ovarian cancer patients completed questionnaires after initial treatment (endometrial, 221 [75%]; ovarian, 174 [71%]) and after 6, 12, and 24 months. SCPs were automatically generated after initial treatment by the oncology providers through the web-based ROGY. Illness perceptions were measured after initial treatment and HRQoL and anxiety and depression after 6, 12, and 24 months.

**Results:**

Structural equation models showed that endometrial cancer patients who experienced more symptoms or concern due to the SCP reported worse social functioning (*β* = − 0.82; *p* = 0.01) and more fatigue, insomnia, pain, and anxiety (*β* = 0.58–0.86, *p* < 0.05) within 12 months after treatment. Ovarian cancer patients who had lower trust that the treatment would cure their disease due to the SCP reported worse emotional functioning 6 months after treatment (*β* = 0.27, *p* = 0.02).

**Conclusions:**

Current results show that SCPs may have negative effects on HRQoL and anxiety in patients who experience more threatening illness perceptions due to the SCP. We should be aware of the potential negative consequences of SCPs.

*Trial Registration* clinicaltrials.gov Identifier: NCT01185626.

## Background

Over the last decade, survivorship care plans (SCPs) have been recommended as a standard of care for all cancer patients. SCPs contain written information to support patients in their physical and psychological challenges in life after treatment [1]. To date, a limited number of randomized controlled trials (RCTs) have been conducted to assess the impact of SCPs on patient reported outcomes [2–7]. As opposed to observational and qualitative studies [8, 9], RCTs failed to identify beneficial effects of SCPs on patient satisfaction with information provision and care, quality of life, or distress [2–5, 7]. However, SCPs may be beneficial for underserved patient populations [6]. The registration system oncological gynecology (ROGY) care trial was the first pragmatic cluster-randomized controlled trial that assessed the impact of automatically generated SCPs, and did find a negative effect on illness perceptions [5, 7].

Illness perceptions are generally defined as a patient’s belief about the disease through cognitive representations, including the perceived impact on life, duration of the illness, experienced symptoms and treatment trust, and also emotional representations, including concern, emotional impact, and personal control over illness [10]. In the ROGY care trial, SCPs caused more threatening illness perceptions: they increased experienced symptoms, emotional impact and concern in endometrial cancer patients [5], and led to lower trust that the treatment would help to cure the disease in ovarian cancer patients [7].

Previous studies in cancer patients show that more threatening illness perceptions are associated with poorer quality of life and more psychological morbidities [11–17] in accordance with Leventhals’ common-sense model of self-regulation (CSM). CSM presumes that individuals who are confronted with a health threat (i.e., cancer diagnosis) form illness perceptions, which impact physical and psychosocial outcomes through coping responses [10, 18]. To support emotional coping, psychological interventions have been developed that aim to decrease psychological distress after an event such as a cancer diagnosis [19]. Exposure therapies, such as psychological debriefing do not seem to decrease psychological morbidity, but may even worsen it due to exacerbation of the symptoms [20]. Similarly, SCPs containing extensive information on the disease and potential side-effects may exacerbate psychological distress and symptoms experienced among cancer patients. Consequently, prior results of the ROGY care trial suggest that SCPs may intervene in the pathway of the CSM by causing more threatening illness perceptions [5, 7], which may in turn affect physical and psychosocial outcomes. However, no evidence exists on the possible causal relationships between SCP provision, illness perceptions, and physical and psychosocial outcomes. It is important to consider the potential negative consequences of threatening illness perceptions due to SCPs before widespread implementation is decided upon.

The aim of the current study is to assess whether SCPs have a negative effect on long-term health-related quality of life (HRQoL), anxiety and depression in patients who experience more threatening illness perceptions due to the SCP. Illness perception scales that have earlier shown to be affected by SCPs [5, 7] (i.e., increased experienced symptoms, concern and emotional impact in endometrial cancer, and lower treatment trust in ovarian cancer) are included in the current analysis. We hypothesize that SCPs have a negative impact on HRQoL, anxiety and depression through more threatening illness perceptions.

## Methods

### Design

The ROGY care trial among endometrial and ovarian cancer patients aimed to assess the longitudinal impact of automatically generated SCPs on patient reported outcomes. A cluster-randomized design was used to avoid potential contamination between the trial arms. Twelve hospitals in the Netherlands were randomly allocated to either ‘usual care’ or ‘SCP care.’ The trial was centrally approved by a Medical Research Ethics Committee [21] and was registered as NCT01185626 in clinicaltrials.gov.

### Participants and recruitment

All newly diagnosed women with endometrial cancer as a primary tumor between April 2011 and October 2012, or ovarian cancer between April 2011 and March 2014, were invited to participate shortly after initial treatment, by means of a letter and an informed consent form, sent directly to the patients’ home address by their own gynecologist. After consent, questionnaires were sent after treatment and follow-up questionnaires were sent at 6, 12, and 24 months after treatment (“Appendix”). Because of the pragmatic nature of the trial, exclusion criteria (i.e., borderline ovarian tumor, undergoing palliative care or unable to complete a Dutch questionnaire) were limited [21]. Earlier analysis showed that 73% of endometrial and 66% of ovarian cancer patients in the SCP care arm reported receipt of an SCP [22]. In the current analysis, all patients of both trial arms were included (intention-to-treat) to reflect real-life clinical practice in which not all patients receive an SCP.

### Randomization and blinding

To prevent imbalance between the trial arms, stratified randomization was used according to whether a hospital has a Gynecologic Oncology Center, and the annual number of endometrial and ovarian cancer patients diagnosed in each hospital. Randomization was performed via a table of random numbers, by an independent researcher blinded to the identity of the hospitals. Patients, but not oncology providers or researchers assessing the outcomes, were blinded to trial assignment [21].

### SCP care versus usual care

In ‘usual care’ hospitals, standard care was provided in accordance to the Dutch follow-up guidelines (http://www.oncoline.nl). In most hospitals, verbal information and the generic brochures of the Dutch Cancer Society were provided [21]. None of the hospitals provided SCPs as developed for this study.

In the ‘SCP care’ hospitals, all oncology providers (gynecologist/gynecologic oncologist and oncology nurses, *N* = 24) attended an instruction evening devoted to when and how SCPs should be provided. They were instructed to provide an SCP to patients at the consultation where the results of histopathology and (adjuvant) treatment plan were discussed, mostly 7–14 days after surgery. If applicable (i.e., if there were any changes in the cancer, treatment or oncology provider), an updated version of the SCP could optionally be discussed in a follow-up consultation. Practical guidelines were given on the components of the SCP that should minimally be discussed with each patient during the consultation (i.e., diagnosis, prognosis, treatment(s), most important side-effects). Because of the pragmatic approach of the trial, care providers in the ‘SCP care’ arm were free to choose whether the gynecologist/gynecologic oncologist, or oncology nurse provided the SCP fitting their clinical practice [21].

### Survivorship care plan

The SCP was based on the Dutch translation of the National Academy of Medicine (NAM) SCP template [1], adjusted to the local situation [21] by a group of gynecologists/gynecologic oncologists, oncology nurses, a radiotherapist, medical oncologist, primary care physician, and patients [21]. The SCP consisted of information on diagnostic tests, type of cancer, stage, grade, and treatments received, and contact details of the hospital and medical specialists. In addition, the SCP contained a tailored follow-up care plan, including detailed information on the most common short- and long-term effects of the treatments received, effects on social and sexual life, possible signs of recurrence and secondary tumors, and information on rehabilitation, psychosocial support, and supportive care services [21]. Texts of the SCP were based on pilot-tested patient education material from the Dutch Cancer Society. In addition, the SCP was pilot-tested on patients with a low/intermediate educational level to ensure that the SCP was understandable.

### Measures

Age, socio-economic status (SES), and clinical data, such as cancer type, cancer stage, and date of diagnosis, were obtained from the Netherlands Cancer Registry (NCR). The NCR routinely collects data on newly diagnosed cancer patients in all hospitals in the Netherlands [23]. SES was based on postal code of the residence area of the patient, combining aggregated individual fiscal data on the economic value of the home and household incomes [24]. SES was categorized into low, medium, or high. Additional socio-demographic information (i.e., marital status, employment status, and comorbidities) was assessed in the first questionnaire. Marital status (‘married/living together’ vs ‘divorced/widowed/never married’) and employment status (‘having a paid job’ vs ‘not having a paid job’) were dichotomized. Comorbidity was assessed by the adapted Self-administered Comorbidity Questionnaire (SCQ), and categorized into no comorbidities, one comorbidity, or more than one comorbidity [25].

The Brief Illness Perception Questionnaire (B-IPQ) was used to assess illness perceptions after initial treatment [26]. The B-IPQ includes eight single-item scales (impact of disease on life, perceived duration of illness, personal control over illness, trust that the treatment would help to cure the illness, experienced symptoms, concern about the illness, understanding of the illness, and emotional impact of the illness). Only the scales that have earlier shown to be affected by SCPs in our trial [5, 7] were used in the analysis, including the amount of symptoms experienced, concerns about the illness, emotional impact of the illness with respect to endometrial cancer, and trust that the treatment would help to cure with respect to ovarian cancer [27]. The latter scale was reversed to ascertain that all B-IPQ scales were one-directional: a higher score indicates more threatening illness perceptions. Test–retest reliability (Pearson correlations 0.42–0.75) was fair [26].

The EORTC QLQ-C30 (version 3.0) was used to assess HRQoL 6, 12, and 24 months after diagnosis [28]. It contains five functional scales on physical, role, cognitive, emotional and social functioning, a global QoL scale; three symptom scales on fatigue, nausea and vomiting, and pain; and six single items. Response scales included: ‘Not at all,’ ‘A bit,’ ‘Quite a bit,’ and ‘Very much,’ except for the global QoL scale, which ranges from ‘Very poor’ to ‘Excellent.’ Latent variables of the scales were defined by the items of each scale [29]. Higher scores on global quality of life and the function scales indicate a better HRQoL, while higher scores on the symptom scales indicate more symptoms. Test–retest reliability was good (Pearson correlations = 0.82–0.91) [30]. Internal consistency of the multi-item scales (Cronbach’s alphas 0.71–0.92) in our study was good.

The Hospital Anxiety and Depression Scale (HADS) was used to assess symptoms of anxiety and depression 6, 12, and 24 months after diagnosis [31]. The HADS assesses separate anxiety and depression scales, which both consist of seven items. All items were scored on a 0–3-point scale, with higher scores indicating more symptoms. Test–retest reliability of the scales (Pearson correlations = 0.86–0.88) was good [31]. Internal consistency of the scales (Crohnbach’s alphas 0.71–0.77) in our study was good.

### Statistical analysis

Statistical analyses were conducted using Statistical Analysis System (SAS) version 9.4. (SAS Institute, Cary, NC, 1999). Differences in characteristics of patients between the trial arms for endometrial and ovarian cancer were compared using independent samples *t* tests for normally distributed continuous variables, Mann–Whitney *U* tests for not normally distributed variables and Chi-square tests for categorical variables. Differences in baseline B-IPQ scores between the trial arms were assessed using Chi-square tests of categorical variables defined by the 25th, 50th, 75th, and 100th percentile scores of the separate B-IPQ scales.

Pearson’s correlation coefficients were computed to assess the correlations between the illness perception scales at baseline and outcomes (HRQoL, anxiety and depression) 6, 12, and 24 months after initial treatment, for endometrial and ovarian cancer separately. *P* values smaller than .05 were considered to be statistically significant.

**Fig. 1:**
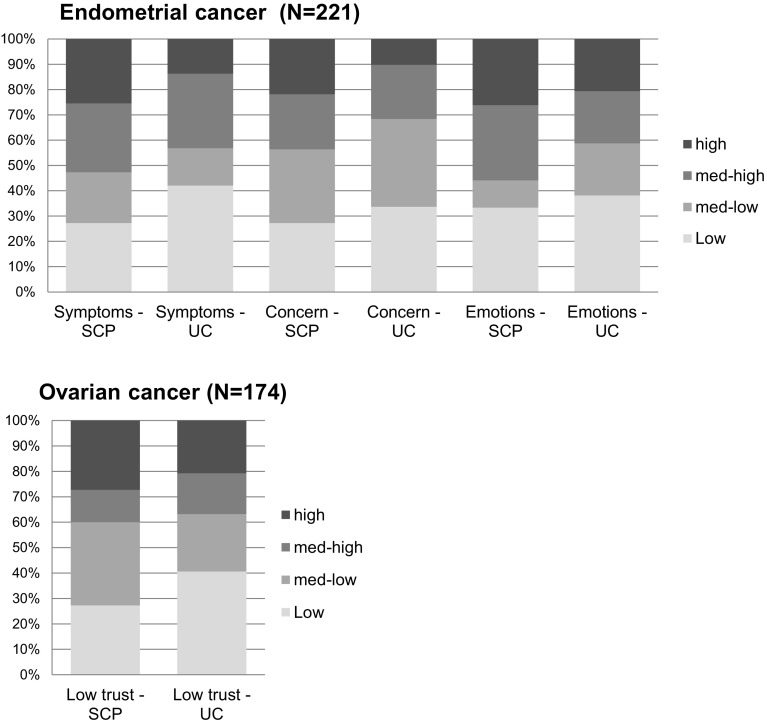
Bar charts of illness perceptions after diagnosis, SCP care (SCP) versus usual care (UC). *Note* only the illness perception items were included that have earlier been associated with trial allocation. High, med-high, med-low, and low illness perception categories were defined by the 25th, 50th, 75th, and 100th percentile scores of each B-IPQ scale separately

Structural equation models (SEM) were used to test the hypothesized causal relationships between trial allocation (SCPs), illness perceptions and the HRQoL, and anxiety and depression scales, with a linear equation system. SEMs are used to assess unobservable ‘latent’ variables by using observed variables, and to assess the relationships between those (observed and latent) variables [32]. Both direct and indirect effects of trial allocation on outcome variables were assessed using the *effpart* statement in the *CALIS procedure* in SAS. Direct effects would indicate an effect of SCPs on the outcome scales in all patients, while indirect effects would indicate an effect of SCPs on the outcome scales in patients who have altered illness perceptions due to the SCP. Statistical power was sufficient to detect indirect effects, but low to detect direct effects [33]. The CALIS procedure was used to define the model paths (i.e., hypothesized relationships between variables). First, simple mediation models were built to assess the direct and indirect effects of trial allocation on the separate outcome scales (HRQoL, anxiety and depression scales) 6, 12, and 24 months after initial treatment. Mediators were the separate B-IPQ scales measured after initial treatment that have earlier shown to be associated with trial allocation (i.e., increased experienced symptoms, concern and emotional impact in endometrial cancer, and lower treatment trust in ovarian cancer; Fig. 1). Models were defined for endometrial and ovarian cancer separately. The paths in each simple mediation model were defined as *trial allocation* ---> [*BIPQ item score after diagnosis*] ---> [*outcome variable at time-point X*]. When outcome scales consisted of multiple items, a latent variable was defined by the items of that scale. When standardized factor loadings of scale items were low (*β* < 0.6), they were removed from the model to obtain a better model fit [34]. When (semi-)complete separation of the outcome scales occurred, no SEM could be determined [35]. Second, the full SEMs were built by entering all significant paths (*p* < 0.05) of the simple mediation models into one model, for endometrial and ovarian separately, and for each time-point separately. Third, the insignificant paths (*p* > 0.05) were removed from the model to obtain a good model fit. Finally, the covariates that were significantly associated with any of the outcome scales were entered into the model. For all SEM models, full information maximum likelihood (FIML) was used, which handles missing data within the model without needing to impute data [36]. Model fit was assessed with the *χ*^2^ statistic, adjusted goodness-of-fit-index (AGFI), Bentler’s Comparative Fit Index (CFI), standardized root mean squared residual (SRMR), and root mean squared error approximation (RMSEA) [37]. Standardized beta coefficients were used to interpret the models, and range from − 1 to 1, in which coefficients closer to zero indicate smaller effects. An increase of 1 standard deviation of the independent variable corresponds to an increase in standard deviation of the dependent variable by the standardized beta coefficient [38]. Standardized beta coefficients of indirect effects can be considered small (0.05–0.10), moderate (0.1–0.25), or large (> 0.25) [38].

## Results

Table 1 shows the clinical and socio-demographic baseline characteristics for both endometrial and ovarian cancer patients in the SCP care and usual care conditions. In endometrial cancer, patients in the ‘SCP care’ took more time after diagnosis to complete the questionnaires than patients in the ‘Usual care’ arm (*p* < 0.01). No differences in baseline characteristics were found between the trial arms in ovarian cancer patients.

**Table 1:** Baseline clinical and socio-demographic characteristics endometrial and ovarian cancer patients, SCP care versus usual care

	Endometrial cancer			Ovarian cancer		
	SCP care (*N* = 119)	Usual care (*N* = 102)	*P* value	SCP care (*n* = 61)	Usual care (*n* = 113)	*P* value*
Age at survey						
Mean (SD)	67.4 (9.1)	67.8 (8.9)	0.71	63.6 (11.2)	64.3 (10.7)	0.67
SES^a^, *n* (%)						
High	43 (36)	36 (35)	0.60	25 (41)	44 (39)	0.12
Intermediate	49 (41)	42 (41)		15 (25)	44 (39)	
Low	21 (18)	22 (22)		12 (20)	18 (16)	
Unknown	6 (5)	2 (2)		9 (15)	7 (6)	
Months since diagnosis, *n* (%)						
Median	2.2	1.5	< **0.01**	3.0	2.4	0.31
< 1	12 (10)	24 (24)		8 (13)	26 (23)	
1–2	40 (34)	46 (45)		18 (30)	39 (34)	
2–3	33 (28)	20 (20)		8 (13)	17 (15)	
> 3	34 (29)	12 (12)		27 (44)	32 (28)	
Comorbidity, *n* (%)						
None	19 (16)	18 (18)	0.53	21 (34)	28 (25)	0.18
1	32 (27)	20 (20)		12 (20)	36 (32)	
2 or more	64 (54)	62 (61)		26 (43)	48 (36)	
Unknown	4 (3)	2 (2)		2 (3)	8 (7)	
Marital status^b^, *n* (%)						
Partner	85 (71)	76 (75)	0.74	48 (79)	82 (73)	0.39
No partner	31 (26)	25 (25)		13 (21)	31 (27)	
Unknown	3 (3)	1 (1)		0 (0)	0 (0)	
Employed, *n* (%)						
Yes	22 (18)	15 (15)	0.40	20 (33)	31 (27)	0.44
No	85 (71)	79 (77)		41 (67)	83 (73)	
Unknown	12 (10)	8 (8)		0 (0)	0 (0)	
FIGO stage, *n* (%)						
I	102 (85)	89 (87)	0.34	21 (34)	31 (27)	0.63
II	6 (5)	1 (1)		7 (11)	9 (8)	
II	9 (8)	9 (8)		23 (38)	50 (44)	
IV	2 (2)	3 (3)		10 (16)	20 (18)	
Unknown	0 (0)	0 (0)		0 (0)	3 (3)	
Treatment, *n* (%)						
Surgery	117 (99)	97 (98)	0.46	54 (88)	104 (93)	0.33
Chemotherapy	6 (5)	12 (12)	0.06	44 (72)	92 (82)	0.13
Radiotherapy	44 (37)	37 (37)	0.99			

*P* values < 0.05 are in bold

^a^Socio-economic status (SES) was based on postal code of the residence area of the patient

^b^Marital status included: *partner* married/living together; *no partner* divorced/widowed/never married. The numbers may not always add up to 100, because percentages have been rounded off to whole numbers

Figure 1 shows the differences in illness perceptions between the SCP care and usual care arms. Significantly more endometrial patients in the SCP care arm compared to the usual care arm reported high experienced symptoms (18% vs 9%, *p* = 0.02) and high concerns about the illness (16% vs 7%, *p* = 0.02). No significant differences between the trial arms were found in emotional impact of the disease (19% vs 14%, *p* = 0.27) in endometrial cancer, or low trust that the treatment would help to cure the illness (16% vs 14%, *p* = 0.60) in ovarian cancer. However i,n earlier multilevel linear mixed model analyses, SCPs significantly increased threatening illness perceptions on these scales [5, 7].

Table 2 shows the correlations between illness perception scales after diagnosis and HRQoL, anxiety and depression after 6, 12, and 24 months, corrected for multiple testing (Bonferroni correction, *α* < 0.003). Consistent with our hypothesis, in both endometrial and ovarian cancer, significant moderate negative Pearson’s correlations were found between B-IPQ items and functioning scales (*r* = − 0.25 to − 0.41, *p* < 0.003). Significant moderate positive Pearson’s correlations were found between B-IPQ items and symptom scales (*r* = 0.27–0.41, < 0.003), and between B-IPQ scales and anxiety and depression (*r* = 0.28–0.46, < 0.003).

**Table 2:** Correlations between illness perception scales after diagnosis and HRQoL, anxiety and depression after 6, 12, and 24 months

	Endometrial cancer									Ovarian cancer		
Illness perceptions after treatment	Symptoms experienced			Concerns			Emotional impact			Lower treatment trust		
Months after treatment, outcome variables	6	18	24	6	18	24	6	18	24	6	18	24

*N*	158	147	128	158	147	128	158	147	128	124	101	75
Global quality of life	− 0.24	− 0.25*	− 0.21	− 0.25*	− 0.29*	− 0.34*	− 0.29*	− 0.29*	− 0.29*	− 0.42*	− 0.40*	− 0.36*
Function scales												
Physical functioning	− 0.22	− 0.19	− 0.24	− 0.14	− 0.21	− 0.19	− 0.18	− 0.22	− 0.18	− 0.24	− 0.16	− 0.16
Role functioning	− 0.06	− 0.20	− 0.28*	− 0.09	− 0.15	− 0.36*	− 0.22	− 0.13	− 0.25	− 0.27	− 0.18	− 0.25
Emotional functioning	− 0.34*	− 0.21	− 0.32*	− 0.32*	− 0.24	− 0.38*	− 0.38*	− 0.29*	− 0.41*	− 0.28*	− 0.15	− 0.27
Cognitive functioning	− 0.17	− 0.07	− 0.25	− 0.14	− 0.17	− 0.28*	− 0.17	− 0.06	− 0.22	− 0.19	− 0.29	− 0.34
Social functioning	− 0.41*	− 0.28*	− 0.32*	− 0.23	− 0.29*	− 0.38*	− 0.33*	− 0.27*	− 0.27*	− 0.16	− 0.07	− 0.26
Symptom scales												
Fatigue	0.36*	0.28*	0.34*	0.26	0.26	0.37	0.35*	0.31*	0.32*	0.26	0.14	0.29
Nausea and vomiting	0.13	0.21	0.24	0.06	0.05	0.14	0.15	0.09	0.12	0.14	0.15	0.33
Pain	0.23	0.17	0.26	0.20	0.23	0.39*	0.22	0.20	0.26	0.28*	0.19	0.33
Dyspnea	0.18	0.19	0.11	0.07	0.14	0.14	0.15	0.15	0.20	0.20	0.02	0.23
Insomnia	0.32*	0.17	0.25	0.23	0.19*	0.28*	0.30*	0.22	0.26	0.31*	0.20	0.18
Appetite loss	0.28*	0.16	0.23	0.18	0.03	0.28*	0.27*	0.14	0.24	0.22	0.09	0.44*
Constipation	0.04	0.03	0.09	0.11	0.02	0.22	0.14	0.08	0.22	0.21	0.21	0.14
Diarrhea	0.18	0.11	0.13	0.15	0.11	0.05	0.21	0.08	0.14	0.06	< 0.01	0.13
Financial difficulties	0.30*	0.20*	0.20	0.17	0.08	0.11	0.21	0.10	0.08	0.16	0.09	− 0.08
Anxiety	0.30*	0.33*	0.34*	0.41*	0.45*	0.46*	0.46*	0.47*	0.45*	0.39*	0.31	0.24
Depression	0.39*	0.28*	0.23*	0.35*	0.35*	0.36*	0.39*	0.33*	0.34*	0.40*	0.25	0.38*

Pearson’s correlations were estimated between illness perceptions measured after diagnosis, and outcome variables after diagnosis and 6, 12, and 24 months after diagnosis. Only the illness perception items were included that have earlier been associated with trial allocation

^*^p < 0.003 (adjusted for Bonferroni correction 0.05/1)

**Fig. 2:**
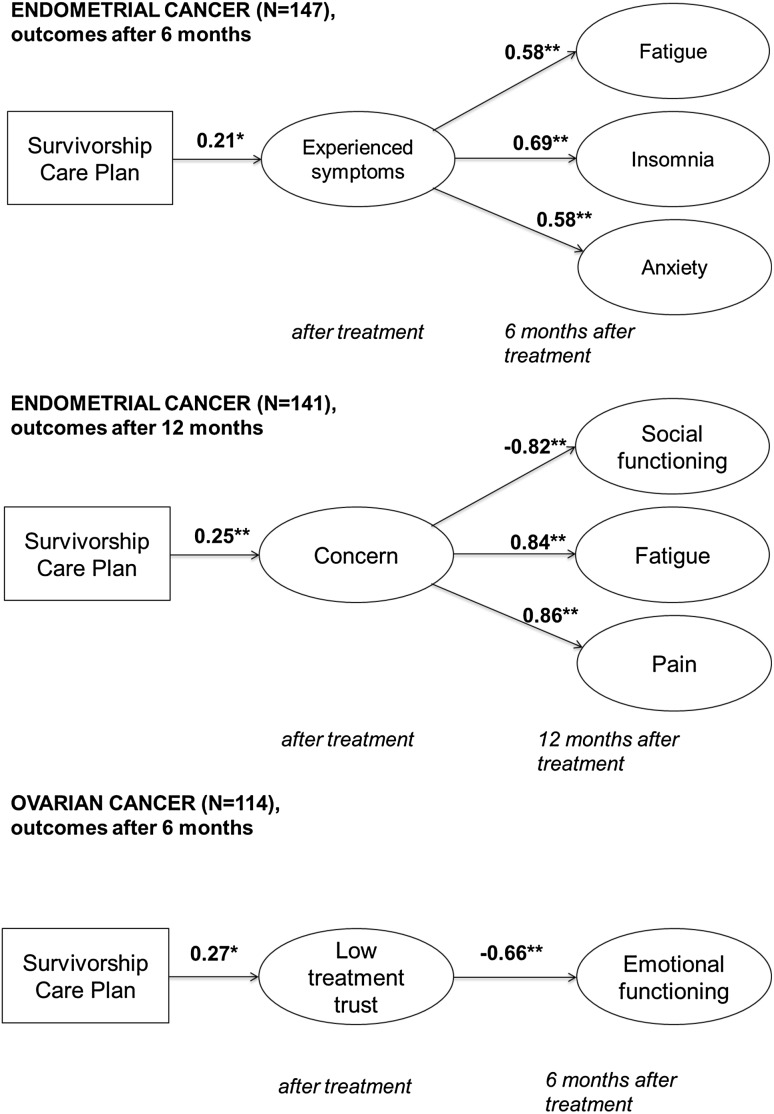
Path diagrams of the final structural equation models, outcomes 6 and 12 months after treatment. *Note* only the significant paths between the intervention (SCP), illness perception items, and outcome scales were entered in this model to obtain good model fit. Standardized coefficients are shown. Standardized beta coefficients were used to interpret the models, and range from − 1 to 1, in which coefficients closer to zero indicate smaller effects. Error terms and covariates in the model (age, FIGO stage, number of comorbidities) have been removed from the figure. ^*^p < 0.05; ^**^p < 0.01

The SEM models showed no direct effects of SCPs on HRQoL, anxiety and depression scales. However, indirect effects through illness perceptions were observed. In endometrial cancer, SCPs indirectly increased fatigue, insomnia, and anxiety after 6 months (standardized, *β* = 0.58, SE = 0.09, *p* < 0.01; *β* = 0.69, SE = 0.08, *p* < 0.01; *β* = 0.58, SE = 0.09, *p* = 0.01), through more experienced symptoms (standardized, *β* = 0.21, SE = 0.09, *p* = 0.02; Fig. 2). Model fit was reasonable to good (AGFI = 0.87; CFI = 0.93; SRMR = 0.07; RMSEA = 0.047 [95% CI = 0.03–0.06]; *χ*^2^ = 87.2, *p* < 0.01) and effect sizes of the indirect effects are moderate (standardized, *β* = 0.12, SE = 0.06, *p* = 0.03; *β* = 0.15, SE = 0.06, *p* = 0.02; *β* = 0.12, SE = 0.05, *p* = 0.03) [38]. Further, SCPs indirectly decreased social functioning after 12 months (standardized, *β* = − 0.82, SE = 0.06, *p* < 0.01), and increased fatigue and pain after 12 months (standardized, *β* = 0.84, SE = 0.05, *p* < 0.01; *β* = 0.86, SE = 0.05, *p* < 0.01), through more concern (standardized, *β* = 0.25, SE = 0.09, *p* < 0.01; Fig. 2). Model fit was good (AGFI = 0.90; CFI = 0.98; SRMR = 0.046; RMSEA = 0.04 [95% CI = 0.00–0.06]; *χ*^2^ = 34.4, *p* = 0.08) and the effect sizes of the indirect effects were moderate (standardized, *β* = − 0.20, SE = 0.07, *p* < 0.01; *β* = 0.21, SE = 0.07, *p* < 0.01; *β* = 0.22, SE = 0.08, *p* < 0.01) [38]. In ovarian cancer, SCPs indirectly decreased emotional functioning after 6 months (standardized, *β* = − 0.66, SE = 0.20 *p* < 0.01), through lower trust that the treatment would help to cure the disease (standardized, *β* = 0.27, SE = 0.12, *p* < 0.05) (Fig. 2). Model fit was good (AGFI = 0.90; CFI = 0.96; SRMR = 0.06; RMSEA = 0.06 [95% CI = 0.01–0.09]; *χ*^2^ = 23.1, *p* = 0.04), and the effect size of the indirect effect was moderate (standardized, *β* = − 0.18, SE = 0.08, *p* = 0.02) [38]. No significant paths in the simple mediation models were found for outcomes after 24 months, for both endometrial and ovarian cancer.

## Discussion

The current study among endometrial and ovarian cancer patients shows that SCPs have a negative impact on long-term HRQoL and anxiety in patients who experience more threatening illness perceptions due to the SCP. Endometrial cancer patients who experience more symptoms or concerns due to the SCP, report worse social functioning and more fatigue, insomnia, pain, and anxiety in the year following treatment. Ovarian cancer patients who have lower trust that the treatment would cure their disease, due to the SCP, report worse emotional functioning 6 months after initial treatment.

Earlier findings from the ROGY Care trial already showed that SCPs increased threatening illness perceptions: higher experienced symptoms, concern and emotional impact in endometrial cancer patients, and decreased trust that the treatment would help to cure the disease in ovarian cancer patients [5, 7]. However, it was yet unclear whether these threatening illness perceptions would deteriorate long-term physical and psychosocial outcomes. Our study confirms earlier findings in cancer patients that more threatening illness perceptions are associated with worse physical and psychosocial outcomes [11–17]. Consequently, our analyses confirmed that threatening illness perceptions due to the SCP led to worse HRQoL and more anxiety. Although no direct effects of SCPs were found, our results support our hypothesis that SCPs have a negative impact on HRQoL and anxiety through more threatening illness perceptions, consistent with Leventhal’s CSM [10].

Illness perceptions that mediated between SCP provision and HRQoL and anxiety were experienced symptoms and concern in endometrial cancer, and low treatment trust in ovarian cancer patients. Possibly, endometrial cancer patients, who are often diagnosed with low cancer stages, may perceive their cancer as a more serious condition due to information provided in the SCP (i.e., the diagnosis and treatments received, possible long-term and late effects, and chance of recurrence) than would otherwise be communicated by the oncology provider. The overall perception of a more serious condition in endometrial cancer patients may have caused higher symptom awareness, more anxiety, and the belief that one is unable to participate in social activities [16]. In ovarian cancer patients, who are more often diagnosed at advanced stages, the SCP led to lower treatment trust, possibly due to information on chance of recurrence in the SCP. Although this information may be realistic, it led to decreased emotional functioning after 6 months, meaning that patients felt more tense, worried, irritable, or depressed. Indeed, fear of recurrence has earlier been found to be most strongly associated with emotional functioning, of all EORTC QLQ-C30 functioning scales [39].

A limitation of the current study is that not all patients in the SCP arm reported receipt of an SCP [22]. A process evaluation of the ROGY care trial showed that ovarian cancer patients, older patients, and patients who have a distressed (type D) personality less often received an SCP [22]. We performed intention-to-treat analysis to reflect real-life clinical practice in which not all patients receive an SCP. Therefore, our results possibly underestimate the effects of SCPs on HRQoL, anxiety and depression in the total population, as patients with a type D personality may be more likely to experience threatening illness perceptions due to the SCP [40]. Further, as shown earlier [7], ovarian cancer patients with higher cancer stages were more often lost to follow-up due to death or ill-health, and were therefore not included in our longitudinal analyses. Therefore, current results in ovarian cancer may represent the healthier patient with lower cancer stages. However, we aimed to minimize selection bias by limiting exclusion criteria and our response rates were relatively high.

Our SEM analyses violated the well-known recommendation of Baron and Kenny’s that a significant relationship between the independent variable (SCPs) and outcome (HRQoL, anxiety and depression) is required, in order to evaluate mediation effects [41]. However, this recommendation has since been criticized [33, 42]. Kenny and Judd argued that sample sizes needed to detect direct effects between independent and dependent variables, should be much larger than to detect indirect effects through mediation [33]. Therefore, our sample sizes of endometrial and ovarian cancer separately, were too small to detect direct effects of SCPs on the outcome scales. Possibly, direct effects of SCPs on the outcome scales would be found in larger sample sizes. An alternative explanation of our findings is the presence of a suppressing mediator, such as coping, which may ameliorate the indirect impact of SCPs on physical and psychological outcomes, while at the same time illness perceptions deteriorate the indirect impact of SCPs on outcomes. Therefore, there may be indirect effects but no direct effects of SCPs on HRQoL and anxiety [42], which would indicate that the impact of SCPs works differently across coping styles.

In earlier publications of the ROGY care trial [5, 7], we argued that information provided in an SCP could be perceived as threatening but may also be *realistic*. Providing patients with honest and realistic information may be considered best to prepare patients for potential consequences of the cancer and cancer treatments, or would encourage patients to find social support to cope with the disease [43]. However, the current study shows that realistic information is not self-evidently beneficial for all patients. Instead, patients may attain worse expectations about negative outcomes such as side-effects or a recurrence, which has shown to potentially cause clinical worsening (“nocebo effect”) [44]. Similarly, psychological interventions that expose individuals to facts and rethinking of an event (i.e., cancer diagnosis and treatments) may not necessarily decrease psychological distress but rather exacerbate symptoms [20]. Possibly, patients with certain personality types or coping styles may be more vulnerable to the harmful effects of exposure interventions such as SCPs. On the other hand, one may argue that patients benefit from having received realistic information on the chance of recurrence in an SCP, after they eventually develop a recurrence. Little is known about the effect of exposure to information about a potentially negative outcome, after the negative outcome has manifested. Unfortunately, numbers of patients with a recurrence in our trial were too small to investigate the impact of SCPs in these patients after diagnosis of a recurrence. Further moderation analysis considering patient characteristics, personality, and coping style could further reveal which patients do, and which patients do not benefit from SCPs.

## Conclusion

In conclusion, no beneficial effects of SCPs on satisfaction with information provision and care in both endometrial and ovarian cancer patients were shown as primary outcomes of our trial [5, 7]. The current study highlights that SCPs may even have negative effects on HRQoL and anxiety in patients who experience more threatening illness perceptions due to the SCP. Therefore, we should be aware of the potential negative consequences of SCPs in some patients. A more tailored approach such as personalized SCPs fitting individual patient’s information needs should be further explored.
